# Outbreak of foodborne gastroenteritis in a senior high school in South-eastern Ghana: a retrospective cohort study

**DOI:** 10.1186/s12889-016-3199-2

**Published:** 2016-07-13

**Authors:** Donne K. Ameme, Holy Alomatu, Albert Antobre-Boateng, Adam Zakaria, Lilian Addai, Klutse Fianko, Bai Janneh, Edwin A. Afari, Kofi M. Nyarko, Samuel O. Sackey, Fred Wurapa

**Affiliations:** Ghana Field Epidemiology and Laboratory Training Programme (GFELTP), University of Ghana, School of Public Health, Legon, Accra, Ghana; Ghana Health Service, Accra, Ghana; Department of Epidemiology and Disease Control, School of Public Health, University of Ghana, Accra, Ghana

**Keywords:** Foodborne, Cohort, Outbreak, Gastroenteritis, *Salmonella*, *Clostridium perfringens*

## Abstract

**Background:**

On 4th February 2015, a group of Senior High School students from Fanteakwa district presented to the emergency unit of the district hospital with complaints of abdominal pain, vomiting and diarrhoea. All the students had eaten from a specific food vendor and had neither eaten any other common meal that day nor the previous day. A foodborne disease outbreak was suspected. We investigated to verify the outbreak, determine its magnitude, identify the source and implement control measures.

**Methods:**

A retrospective cohort study was conducted. We reviewed medical records and interviewed patrons of the food vendor. We collected data on age, sex, signs and symptoms, date of illness onset, date of admission, date of discharge, treatments given and outcome. A case of foodborne disease was any person in the school with abdominal pain, vomiting and or diarrhoea from 4th to 11th February 2015 and had eaten from the food vendor. We conducted active case search to identify more cases. We conducted environmental assessment and collected clinical and food samples for laboratory testing. Descriptive and inferential statistical analyses were performed using Stata 12.0.

**Results:**

A total of 68 cases were recorded giving overall attack rate of 35.79 % (68/190) with no death. Of these, 51.47 % (35/68) were males. Mean age of case-patients was 17.8 (standard deviation +/-1.62). The index case, a 17-year-old female student ate from the food vendor on 4th February at 9:00 am and fell ill at 3:40 pm later that day. Compared to those who ate other food items, students who drank water from container at the canteen were more likely to develop foodborne disease at statistically significant levels [RR = 2.6, 95 % CI = (2.11–3.15)]. *Clostridium perfringens (C. perfringens)* and *Salmonella species (Salmonella spp)* were isolated from water and stew respectively. Clinical features of case-patients were compatible with both organisms.

**Conclusion:**

A foodborne gastroenteritis outbreak occurred in a Senior High School in Fanteakwa District from 4th to 7th February 2015. The most probable aetiologic agent was *C. perfringens* with contaminated water at canteen as the vehicle of transmission. Concurrent *Salmonella spp* infection could not be ruled out. Rapid outbreak response helped in controlling the outbreak.

## Background

Foodborne diseases are common yet preventable burden of diseases globally [1]. They are acquired by the consumption of food contaminated with toxins, viruses, bacteria or parasites [2]. Though a growing public health concern worldwide because of the associated high levels of morbidity and mortality [3], foodborne diseases are markedly underreported in most countries [4, 5]. This is particularly so in developing countries, where systematic foodborne disease surveillance and epidemiological studies are seldom undertaken [6]. Globally, foodborne and waterborne diseases kill an estimated 2.2 million people annually [7]. In Ghana, foodborne diseases are hardly reported and outbreaks therefore go undetected partly because of non-existence of a dedicated foodborne disease surveillance system. Despite this, a few foodborne disease outbreaks have been investigated in the Eastern Region [8, 9].

A myriad of aetiologic agents are responsible for foodborne diseases. *Clostridium perfringens* (*C. perfringens)* and *Salmonella species (Salmonella spp)* foodborne diseases are among the commonest foodborne diseases worldwide [10–12]. Though they share similar symptoms like diarrhoea and abdominal pain, fever is characteristic of Salmonella infection whilst vomiting is rare in *C. perfringens* infection. *C. perfringens* foodborne disease is one of the widespread foodborne diseases documented in industrialized nations and yet underreported because of mild nature and relatively shorter duration of its symptoms [10, 12]. *Salmonella spp* foodborne diseases share similar characteristics including underreporting, even though they may occasionally cause serious extra-intestinal complications [2, 13, 14]. When reported, suspected foodborne diseases case-patients are not routinely tested to confirm the offending pathogen or its enterotoxins [15, 16].

Most foodborne disease outbreak investigations have implicated a single pathogenic organism [8, 17–19]. However, multi-pathogen related foodborne outbreaks are presumed to be relatively common. Dual pathogen foodborne disease outbreaks linked to *Salmonella* and *Campylobacter species* have been reported [20, 21]. In Ghana, Opare et al. [9] have reported a gastroenteritis outbreak in a Senior High School in which two pathogens were implicated. We report another mixed-pathogen related foodborne disease outbreak in a Ghanaian Senior High School.

On 4th February 2015, a group of students from a Senior High School in Fanteakwa district were brought to the emergency unit of the district hospital with complaints of abdominal pain, vomiting and bloody mucoid diarrhoea. All the students had eaten from a specific food vendor. Apart from that, they had not taken any meal in common on that day or the previous day. Other students who ate from this food vendor the following day also complained of similar symptoms. The food, which was cooked at the food vendor’s premises, was sold to other members of the community before being taken to the school canteen where the bulk of it was sold. The hospital authorities suspected a foodborne disease outbreak and notified the Eastern Regional Health Directorate through the District Health Management Team (DHMT), which constituted an outbreak investigation team and immediately commenced investigation into the outbreak. We investigated to verify the suspected outbreak, determine its magnitude, identify the source and implement control and preventive measures.

## Methods

### Outbreak setting

The outbreak investigation was conducted at Begoro, the capital of the Fanteakwa District from 7th to 11th February 2015. The district is one of the 26 districts in the Eastern Region of Ghana and has a population of 118, 029 with 58,660 (49.7 %) males and the sex ratio of 98.8 [22]. The district has seven sub-districts, which are served by 14 public and private health facilities including the district hospital. There are 13 functional Community-based Health Planning and Services (CHPS) zones, which are the lowest level of healthcare delivery. The last time an outbreak occurred in the district was in August 2014 where 22 cases of cholera were reported with no deaths. The Senior High School where the outbreak occurred has both day and boarding facilities and student population of 1757 comprising 933 males. The teaching and non-teaching staff are 185. The boarders are fed from the school kitchen whilst all students and staff patronize private food vendors at the school canteen. Private food vendors prepare food from their various premises and transport it to the school canteen for sale. Sometimes, the food prepared by these food vendors is sold to community members as well. A school nurse mans the school’s infirmary, where minor aliments are managed. The source of water for all activities in the school is a mechanized borehole, which supplies storage tanks placed at vantage points in the school. Community water source is a well located at the outskirts of the town.

### Data collection

We interviewed the district health officials and school authorities to obtain information on the nature of the situation. We reviewed records at the District Health Directorate, the district hospital emergency and outpatient departments as well as the wards. Data was abstracted on age, sex, signs and symptoms, date of illness onset, date of admission, date of discharge, treatments given and outcome. We visited the affected case-patients who were still on admission at the hospital. We assessed the management of the affected case-patients at the school and the health facilities. We interviewed students, community members and some of the affected case-patients. We defined a case of foodborne disease as any person in Begoro presenting with abdominal pain, vomiting and or diarrhoea from 4th to 11th February 2015 and had eaten from the food vendor. We conducted active case search in the community, the Senior High School and other health facilities to identify more cases. We updated the line list with the new cases identified. We inspected the food vendor’s premises to assess the food preparation, storage and transportation process. The food vendor was examined for open wounds, skin infections and her health certificate inspected. We held a meeting with students and staff to educate them on personal and environmental hygiene and the causes and prevention of foodborne disease. A meeting was also held for all the food vendors to educate them on proper food handling, personal and environment hygiene. All food vendors had been stopped from cooking for the students until the investigation was concluded.

### Laboratory investigations

Before arrival of the investigation team, local health officials had taken stool sample from one of the case-patients to the Eastern Regional Hospital laboratory for analysis. We took stool, throat swab and nasal swab specimens from the affected food handler for laboratory analysis at the Eastern Regional Hospital laboratory. Samples were transported on appropriate transport media via a triple packaging system to the laboratory within four hours of collection. Samples were tested for enteric pathogens using standard protocols for parasite and bacteria detection.

Most of the leftover food from the food vendor had been discarded. Only stew and “shitor” were available at the food vendor’s premises. We took samples of the leftover food items. We also took water samples from the school compound, community water source and the food vendor’s premises. All food and water samples were collected into sterile containers under aseptic conditions and transported on ice packs to the Food and Drugs Authority (FDA), Accra for laboratory analysis using International Organization for Standardization (ISO) methods for specific food items.

### Environmental survey

An environmental survey of the school compound was conducted to assess the general sanitation of the school. We inspected the schools kitchen, pantry and dining hall and canteen. We also inspected their places of convenience and refuse disposal sites. We inspected their sources of water.

### Surveillance

We assessed the surveillance of foodborne disease and other unusual events in the district. We evaluated the timeliness of detection and reporting of the cases as well as analysis of the data collected.

### Data analysis

We performed descriptive analysis of the outbreak data by person, place and time. Univariable analysis was done by expressing categorical variables as frequencies and relative frequencies. Continuous variables were expressed with appropriate measures of central tendency and dispersion. Based on information obtained from the investigation, we hypothesized that the outbreak was associated with consumption of a particular food item from the food vendor. To test this hypothesis, we conducted a retrospective cohort study where we compared dichotomized qualitative exposure variables (food items eaten) among ill and non-ill students who had eaten from the food vendor. We calculated overall attack rate, sex specific attack rates, food specific attack rates and risk ratios (RR). We drew an epidemic curve to show the magnitude and the course of the illness. In bivariable analysis, we calculated the RR and their corresponding 95 % confidence intervals (CIs) associated with consumption of each food item. We determined variables significantly associated with foodborne disease at significance level of 0.05. Variables significantly associated with foodborne disease in the bivariable analysis as well as those found to have yielded pathogens during laboratory analysis were put in a modified Poisson multivariate model with log link using robust error variances to generate adjusted RR and their CIs. Data was analysed using Stata version 12.0.

## Results

### Descriptive epidemiology

A total of 68 cases were recorded out of the 190 persons who ate from the food vendor. Of the case-patients, 51.47 % (35/68) were males. The mean age of case-patients was 17.8 (standard deviation +/-1.62). The ages of the case-patients ranged from 14 to 24 years. The most affected ages were 17 and 18 years with 22.06 % (15/68) and 33.82 % (23/68) cases respectively. Of the case-patients, 31.34 % (21/68) were day students. The overall attack rate was 35.79 % (68/190) with no death. Sex specific attack rates were 34.31 % (35/102) and 37.50 % (33/88) for males and females respectively. The index case was a 17-year-old form two female student of the Senior High School who took “waakye” and stew on 4th February at 9:00 am from the food vendor and presented later in the day at 3:40 pm with abdominal pain diarrhoea and vomiting. Twenty (57.14 %) of her 35 colleagues who ate “waakye” from the same food vendor that very morning complained of abdominal pain and were all taken to the hospital. The food vendor sold food to some community members prior to transporting food to the school canteen. None of the 24 community members who bought food from the food vendor on the 4th and 5th February 2015 experienced abdominal pain, vomiting or diarrhoea.

The commonest symptoms experienced by the case-patients were abdominal pain and diarrhoea occurring in 47 (74.60 %) and 28 (44.44 %) respectively. Twenty six (41.275 % had fever 14 (22.22 %) had bloody diarrhoea (Table 1).

**Table 1:** Frequency of signs and symptoms among cases (*n* = 68)

Signs and symptoms	Number of cases	Percentage (%)
Abdominal pain	47	74.60
Diarrhoea	28	44.44
Fever	26	38.24
Nausea	21	30.88
Vomiting	14	22.22
Bloody diarrhoea	14	22.22
Headache	4	5.88

**Fig. 1:**
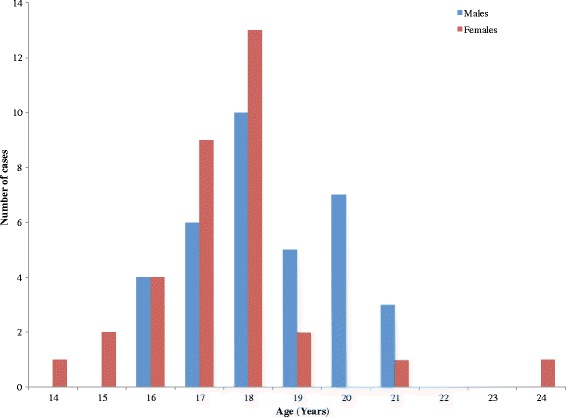
Age and sex distribution of foodborne disease in a Senior High School, Fanteakwa District, February 2015

Among the ages with the highest frequencies (17 and 18 years), more females were affected than males (Fig. 1).

**Fig. 2:**
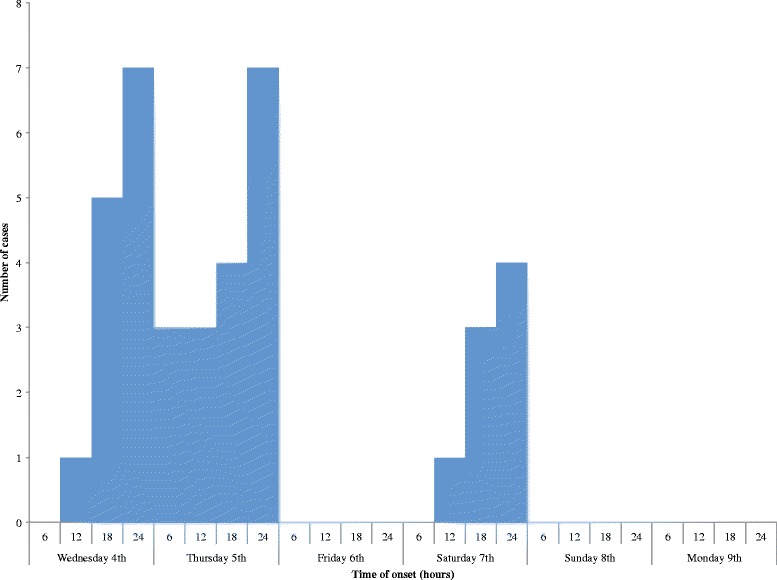
Epidemic curve of the foodborne disease outbreak in a Senior High School, Fanteakwa District, February 2015 (Time of onset was unknown for all case-patients whose illness started on Friday 6th February)

The epidemic curve is suggestive of a common source outbreak with intermittent exposure. The outbreak started on the Wednesday 4th of February and peaked in the last six hours of the day. This pattern was repeated on Thursday, 5th of February. On Friday 6th February, no case was reported. Few cases occurred on Saturday 7th February. No new cases were reported on Sunday and Monday (Fig. 2). The median incubation period was nine hours (range: 1–15).

### Inferential analysis

Out of the food items and water consumed from the food vendor, three items were significantly associated with development of foodborne disease (Table 2). Compared to those who ate other food items on 4th February, students who drank water from container at the canteen were thrice more likely to develop foodborne disease at statistically significant levels [RR = 2.6, 95 % CI = (2.11–3.15)] at statistically significant levels. No other food item was positively associated with development of foodborne disease. Eating egg from the food vendor and drinking sachet water from the canteen on 4th February were protective of foodborne disease [RR = 0.6, 95 % CI = (0.33–0.96)] and [RR = 0.4, 95 % CI = (0.32–0.47)] respectively (Table 2). None of the food items taken on 5th February were however significantly associated with foodborne disease (Table 3).

**Table 2:** Food specific attack rates of foodborne disease in a Senior High School, Fanteakwa District, 4th February 2015 (*n* = 158)

Food item	Exposed			Unexposed			Risk ratio	95 % Confidence Interval
	Ill	Total	Attack rate	Ill	Total	Attack Rate		
Water from container	2	2	1.00	59	152	0.39	2.6	2.11–3.15
Egg	12	47	0.26	50	111	0.45	0.6	0.33–0.96
Sachet water	59	152	0.39	2	2	1.00	0.4	0.32–0.47
“Shitor”	52	126	0.41	10	32	0.31	1.3	0.76–2.30
Pasta	36	80	0.45	26	78	0.33	1.4	0.91–2.01
“Waakye”	62	158	0.39	0	0	0.00	*	*
“Gari”	36	91	0.40	26	67	0.39	1.0	0.69–1.51
“Wele”	4	10	0.40	58	148	0.39	1.0	0.47–2.23
Salad	29	81	0.36	33	77	0.43	0.8	0.57–1.23
Fish	14	43	0.33	48	115	0.42	0.8	0.48–1.26
Meat	3	14	0.21	59	144	0.41	0.5	0.19–1.45
Stew	25	60	0.42	37	98	0.38	1.1	0.75–1.63

*The presence of zeros in some comparisons made it impossible to calculate the RR and or CI

“Waakye”: Boiled rice and beans

“Gari”: Grated cassava that is fried and eaten raw, or served with other food

“Wele”: Cowhide cooked and usually served in sauce

“Shitor”: Hot black pepper sauce

**Table 3:** Food specific attack rates of foodborne disease in a Senior High School, Fanteakwa District, 5th February 2015 (*n* = 78)

Food item	Exposed			Unexposed			Risk ratio	95 % Confidence Interval
	Ill	Total	Attack rate	Ill	Total	Attack Rate		
Water from container	0	0	0.00	14	75	0.19	*	*
Egg	4	25	0.16	12	53	0.23	0.7	0.25–1.97
Sachet water	15	77	0.19	0	0	0.00	*	*
“Shitor”	10	61	0.16	6	17	0.35	0.5	0.20–1.09
Pasta	11	51	0.22	5	27	0.19	1.2	0.45–3.00
“Waakye”	16	78	0.21	0	0	0.00	*	*
“Gari”	9	51	0.18	7	27	0.26	0.7	0.28–1.63
“Wele”	0	6	0.00	16	72	0.22	0.0	*
Salad	10	46	0.22	6	32	0.19	1.2	0.47–2.87
Fish	1	20	0.05	15	58	0.26	0.2	0.03–1.37
Meat	3	16	0.19	13	62	0.21	0.9	0.29–2.76
Stew	7	35	0.20	9	43	0.21	1.0	0.40–2.31

*The presence of zeros in some comparisons made it impossible to calculate the RR and or CI

After adjusting for stew, sachet water and egg, water from container remained significantly associated with development of foodborne disease [adjusted RR = 2.9, 95 % CI = (1.96–4.26)]. Egg was however protective from development of foodborne diseases at statistically significant level [adjusted RR = 0.5, 95 % CI = (0.32–0.91)] (Table 4).

**Table 4:** Multivariable analysis showing food item independently associated with foodborne disease in a Senior High School, Fanteakwa District, February 2015

Food item	Adjusted Risk Ratio	95 % Confidence Interval	*p*-value
Water from container	2.9	1.96–4.26	<0.001
Egg	0.5	0.32–0.91	0.02
Stew	1.2	0.80–1.72	0.41

### Laboratory results

No enteric pathogen was isolated from the stool sample taken by the local health officials. Additional stool samples taken from the affected case-patients who were on admission were negative for cysts, intestinal parasites and enteric pathogens such as *Samonella, Shigella, Clostridium, Escherichia* and *Campylobacter species.* Nasal and throat swabs from the food vendor did not culture *Staphylococcus* or *Streptococcus species*. Stew and water samples taken from the food vendor’s premises isolated *Salmonella spp* and *C. perfringens* respectively. Aerobic plate count of the water sample from the school and community water source exceeded the acceptable limits but did not culture *C. perfringens* or *Escherichia coli.* With the exception of “shitor” from the food vendor that met ISO specifications, all the other items were unacceptable for human consumption under ISO conditions.

### Coordination

The response to the outbreak was through multi-sectoral collaboration. The DHMT constituted a team made up of the members of the DHMT, the hospital staff, the District Assembly and the Ghana Education Service. The investigation team was supported by the Eastern Regional Health Directorate and staff of FDA. The Senior High School authorities also collaborated with the investigation team by helping in the organization of meetings and assisted the team in conducting the interviews. The students were promptly taken to the hospital and the regional health authorities were duly notified.

### Case management

The affected students were managed at the district hospital and an adjoining clinic. The case-patients were managed on Oral Ciprofloxacin, Metronidazole and Oral Rehydration Salt (ORS). Those admitted or detained were given intravenous Metronidazole, Ciprofloxacin and Intravenous Fluids (IVF). Blood and vomitus samples were not taken at both health facilities where the students reported. Stool sample was taken from only one of the case-patients at the district hospital. The school’s infirmary did not have any ORS and therefore no student was given first aid prior to referral to the hospital. However, all the students were promptly referred to the hospitals. All the case-patients were discharged by 8th February 2015.

### Surveillance

During the outbreak investigation, data was collected through passive and active surveillance. Surveillance for diarrhoeal diseases has been enhanced in the school. The staff, students and food vendors were educated on the causes and prevention of gastroenteritis. They were sensitized on regular hand washing with soap and running water. The health facilities have also been placed on high alert for diarrhoeal diseases and any unusual events.

### Environmental issues

The school environment was generally clean. However, there were two open refuse dumps near the dormitories. The environment around the school kitchen was bushy. The school canteen was closed down at the time of the investigation. The environment at the canteen looked clean but there were stray animals (goats) in the vicinity and on the school compound. Some of the kitchen staff were not appropriately dressed especially those at the pantry. They were not wearing aprons and some had not covered their heads during food preparation.

Environmental assessment of the food vendor’s vicinity revealed some infractions in food safety practices. Though the food vendor’s vicinity looked quite clean, the kitchen was unkempt. Cooking utensils were left on the floor uncovered and exposed to animals. Some of the foodstuff and food leftover were stored in a wooden cupboard. The food vendor fetches water from the community well and stores in poorly covered barrels for domestic activities. The food vendor denied having any assistance that helped in the food production process. She also denied any recent history of hospitalization, diarrhoea prior to and during the period of the outbreak investigation. She was an asymptomatic, afebrile and healthy looking young lady who did not have any open wounds or visible skin infections. She also did not possess a valid health certificate.

## Discussion

The occurrence of food and water borne disease outbreaks in school settings are not rare events [9, 23, 24] and has been reported in developed countries [25, 26]. Aetiologic agents implicated in these outbreaks vary. Our study illustrates a dual-pathogen related outbreak, which is less commonly reported in many foodborne outbreak investigations probably because of the fact that investigations are halted once an offending pathogen is identified [20]. Based on our findings, though *C. perfringens* and *Salmonella spp* were isolated from water and food respectively, epidemiological evidence points more to *C. perfringens* as the most likely aetiologic agent of this outbreak. This is because *C. perfringens* was isolated from water, which was the most likely vehicle of transmission. However, this conclusion is not without challenge as some of the signs and symptoms exhibited by the case-patients were peculiar to *Salmonella* infection. The challenge of concluding on a single pathogen as the aetiologic agent of an outbreak has been acknowledged by [27] and observed in another study [28] where clinical, laboratory and epidemiological features were used to differentiate likely aetiology of foodborne diseases. Mixed-pathogen related foodborne disease outbreaks, though rarely documented, have been reported in Ghana [9] and elsewhere [20, 21, 29]. In the later studies, a single food item was contaminated by multiple bacterial pathogens contrary to multiple pathogens found in different food items, as was the case in our study.

The epidemiological and laboratory characteristics of this outbreak make it difficult to pinpoint a single aetiologic agent at first glance. Though water from container at the canteen was epidemiologically implicated with a significant positive association with development of foodborne disease and reinforced by laboratory isolation of *C. perfringens* from water, this was insufficient to confirm *C. perfringens* as the aetiologic agent of the outbreak. Though some authors have suggested that *C. perfringens* foodborne disease can be confirmed by detecting the organism in suspected food that was consumed [30], others have suggested detection of enterotoxins from stool samples of case-patients as a definitive method of implicating the organism as the aetiologic agent [2, 26, 28, 31]. Lack of isolation from the stool samples of case-patients and unavailability of serotyping techniques to demonstrate enterotoxin-producing strains limit the confirmation of *C. perfringens* as the aetiologic agent. This is particular so when *Salmonella spp* which was also isolated from leftover stew had similar clinical presentation and course as observed among the case-patients. Although, stool samples of case-patients were not collected for laboratory analysis precluding comparison with isolated pathogens from the leftover food, the clinical, laboratory and epidemiological analysis points to both *C. perfringens* and *Salmonella spp* as the most likely causative organisms. Similarities in clinical course and characteristics of outbreaks related to these two organisms as well as failure to demonstrate enteric pathogens in stool samples of case-patients made it challenging to single out any of these two organisms as the causative agent. Although *Salmonella spp* was isolated from the leftover stew; epidemiologically, stew did not show any significant positive association with development of foodborne disease. However, symptoms exhibited by the case-patients were analogous to Salmonellosis. The presence of fever in more than a third of the case-patients and bloody diarrhoea in more than a fifth of case-patients were compatible with Salmonellosis although predominance of diarrhoea and abdominal pain as symptoms strongly correlates with disease caused by either of the two organisms. Based on suggestions for discriminating between *C. perfringens* and other organisms using clinical features [27], very few (less than a quarter) of the case-patients experiencing vomiting in our study is somewhat compatible with *C. perfringens* related outbreak. Bennet et al. [27] and Wahl et al. [30] reported vomiting as a rare symptom in *C. perfringens* infections. The median incubation period of nine hours and relatively short duration of illness were all in favour of both organisms. The incubation period for *C. perfringens* and *Salmonella spp* foodborne diseases are 6–24 h [2, 10, 12] and 6–72 h [2] respectively. It is worth noting that differences in the doses of inoculum ingested, susceptibility of the individuals and the quality of the information may explain the variability in incubation period [17] and the clinical presentation of the disease. Also the duration of illness for each case-patient was not assessed.

Contamination of the water source and stew from the food vendor’s premises with *C. perfringens* and *Salmonella spp* respectively indicates improper food handling practices. Lack of refrigerator to store leftover food items could also contribute to this. In this outbreak, the food vendor had no valid health certificate, which is unacceptable for food production industry. Gaps in enforcement of food safety regulations may account for this observation. More stringent measures are therefore important to prevent future occurrences.

With regards to the possible contributory factors to food contamination, major lapses in food safety practices were observed. The lack of an assistant for the food vendor presents a potential for errors in the food production process. The preparation, transportation, sale of food and washing of dishes by a single person to a large number of patrons is unsafe and has the potential for improper food handling and likely contamination. Presence of animals in the kitchen of the food vendor, and lack of possession of valid health certificate were contraventions of food safety regulations that could compromise the quality of food and therefore needed attention.

A key strength of this outbreak investigation is the multi-sectoral collaboration and swiftness of response. However, a major limitation is the fact that limited clinical samples from affected case-patients were taken for laboratory analysis. Local health officials collected stool sample from only one case-patient and all except two case-patients were discharged prior to arrival of the investigation team. This was a missed opportunity for isolating offending pathogens or their enterotoxins from stool. Blood and vomitus samples were not collected for laboratory analysis. In addition, is the lack of advanced laboratory testing for identification of the specific types of organisms either through enterotoxin determination or serotyping. Worthy of note is the fact that identification of pathogens in stool in itself is insufficient to confirm the aetiology of the outbreak as some enteric pathogens have been found in the stool of healthy individuals. *C. perfringens* spores in particularly have been demonstrated in high numbers in the stool of healthy individuals [10] and foodborne disease caused by *C. perfringens* could be diagnosed by quantitative culture of implicated food items or positive faecal enterotoxins [32]. Also, isolating homogeneous strains of an organism or enterotoxin from stool samples of two or more ill persons will suffice for implicating any of these organisms as the aetiologic agent of the outbreak [26, 33]. Furthermore, most of the food items served by the food vendor were not available for testing. The laboratory isolation of the organism was done only in the stew and water, and not in stool sample posing a challenge in linking organisms isolated from food samples with enteric organisms from stool. Establishing that isolates from food and stool samples of case-patients are enterotoxin producing strains as well as matching of strains from food and case-patients have been highly recommended in outbreak settings [10]. These, however, were not done in our investigation because of lack of isolation of enteric pathogens in stool probably as a result of collection of inadequate number of stool samples by local health authorities or antibiotic treatment in the case of the case-patients whose stool samples were collected whilst on admission. Consideration should also be given to the limited routine diagnostic capacity in resource-poor settings. However, aetiologic agents of foodborne outbreaks have been confirmed without laboratory examination of stool specimen [30] or food samples [28, 31]. Also worth consideration is the fact that the clinical samples and food items were not tested for presence of viruses.

The inspection of the food vendor’s environment was also done at the time no food production was taking place and therefore provided an incomplete picture of the food storage, preparation and transport on the particular day of the outbreak. Another limitation is the possibility of recall bias where case-patients were likely to recall their experiences differently from non case-patients. Closely related to this is the difficulty of the case-patients in recalling the exact time of their illness onset. This created the impression as though no cases occurred on Friday 6th February where in fact, several case patients had their illness onset on that day. This therefore gave a distorted picture of the epidemic curve. Misclassification of non case-patients as case-patients could also not be ruled out because of the highly sensitive nature of our case definition. These notwithstanding, the study provides useful lessons in responding to outbreak investigations.

## Conclusions

A dual-pathogen related foodborne gastroenteritis outbreak occurred in a Senior High School in Fanteakwa District, from the 4th to the 7th February 2015. The epidemiological evidence is strongly suggestive of *C. perfringens* as the aetiologic agent but co-infection with *Salmonella spp* could not be ruled out. The most likely vehicle of transmission was contaminated water used by food vendors at the school canteen on 4th February. Rapid response to the outbreak by case management, withdrawal of food vendors from the school canteen and health education helped in controlling the outbreak and preventing more cases. Strengthening of laboratory and human resource capacity as well as formal recognition of street food industry through formulation and rigorous enforcement of appropriate food safety regulations are justified measures to prevent future occurrences. These measures, when complemented with regular education, medical examination and supervision of food handlers have the potential of curbing foodborne diseases particularly those related to street food vending.

## Abbreviations

CHPS, Community-based Health Planning and Services; CI, confidence interval; DHMT, District Health Management Team; FDA, Food and Drugs Authority; ISO, International Organization for Standardization; IVF, Intravenous Fluid; ORS, Oral Rehydration Salt; RR, risk ratio
